# Hockey STAR: A Methodology for Assessing the Biomechanical Performance of Hockey Helmets

**DOI:** 10.1007/s10439-015-1278-7

**Published:** 2015-03-30

**Authors:** Bethany Rowson, Steven Rowson, Stefan M. Duma

**Affiliations:** Department of Biomedical Engineering and Mechanics, Virginia Tech, 313 Kelly Hall, 325 Stanger Street, Blacksburg, VA 24061 USA

**Keywords:** Concussion, Linear, Rotational, Acceleration, Risk, Impact

## Abstract

Optimizing the protective capabilities of helmets is one of several methods of reducing brain injury risk in sports. This paper presents the experimental and analytical development of a hockey helmet evaluation methodology. The Summation of Tests for the Analysis of Risk (STAR) formula combines head impact exposure with brain injury probability over the broad range of 227 head impacts that a hockey player is likely to experience during one season. These impact exposure data are mapped to laboratory testing parameters using a series of 12 impact conditions comprised of three energy levels and four head impact locations, which include centric and non-centric directions of force. Injury risk is determined using a multivariate injury risk function that incorporates both linear and rotational head acceleration measurements. All testing parameters are presented along with exemplar helmet test data. The Hockey STAR methodology provides a scientific framework for manufacturers to optimize hockey helmet design for injury risk reduction, as well as providing consumers with a meaningful metric to assess the relative performance of hockey helmets.

## Introduction

Football is often the focal point of concussion research because of its popularity and the high incidence of concussions associated with it; however, the rate of concussion is higher in ice hockey.^8^,^30^ Moreover, it is the most common injury for women’s collegiate ice hockey, and the second most common for men’s.^1^,^2^ The current helmet safety standards for hockey helmets have changed little over the past 50 years when they were created to reduce the incidence of serious head injuries and deaths.^40^ The first hockey helmet standards were instituted by the Swedish Ice Hockey Association (SIA) in 1962. Shortly thereafter, US and Canadian organizations developed similar standards. Today, most hockey helmets bear stickers representing certification by 3 different organizations: the Hockey Equipment Certification Council (HECC), the Canadian Standards Association (CSA), and the International Organization for Standardization (ISO) represented by a CE marking. These standards all have similar pass/fail criteria that were implemented to reduce the risk of catastrophic head injuries.

Recently, concussion has gained national attention and become a research priority as the incidence of injury rises and concerns about the long-term effects of repeated mild injury are brought to light.^10^,^21^,^30^,^37^,^41^,^42^ Many strategies have been employed in attempts to decrease the incidence of concussion, such as rule changes, education programs, legislation, and improvements in protective equipment.^5^,^36^ Examples of rule changes designed to reduce injuries include fair-play and body-checking rules, which are implemented in some ice hockey leagues. Studies have shown a reduction in the incidence of more serious injuries including concussions when these rules are in place.^34^,^46^ Education programs such as the Centers for Disease Control and Prevention’s “HEADS UP” on concussion initiative and the Hockey Concussion Education Project (HCEP) were developed to help educate coaches, players, and their parents on preventing, identifying, and responding appropriately to concussions.^9^,^12^,^14^–^18^,^32^ Although most states in the US now have concussion laws in place, it is unclear at this time how effective they are.^5^ These laws usually focus on education, removal from play, and approval required for return to play.

There is currently no objective information available to consumers on which hockey helmets provide better protection against serious, as well as milder, head injuries like concussions. Prior to the development of the Football Summation of Tests for the Analysis of Risk (STAR) Evaluation System in 2011, this information was not available for football helmets either.^48^ Since the first set of helmet ratings using this evaluation system was released, the number of helmets receiving the highest rating possible of 5 stars has risen from just one to a total of 12 helmets in 2014.^51^ In the past, there were no conclusive studies on the effectiveness of different helmet types in reducing concussions on the field.^5^,^36^ However, recent research has demonstrated that the risk of concussion on the field is lowered with a helmet that better reduces head accelerations upon impact.^53^

Football STAR was developed based on two fundamental principles. The first is that the tests performed are weighted based on how frequently a similar impact would occur on the field during one season of play.^48^ The second is that helmets that decrease acceleration decrease the risk of concussion. There are a number of concussion risk functions that have been developed to define probability of concussion as a function of linear head acceleration, angular head acceleration, or both.^19^,^31^,^44^,^48^,^49^,^52^,^58^ Debates over the mechanisms of brain injury and the ability of metrics that include linear or angular head acceleration to predict injury risk are long-standing.^27^,^31^ Numerous studies have attempted to differentiate the effects of linear and angular head accelerations on brain injury and determine if one or the other is more likely to result in concussion.^22^,^43^,^55^ Current metrics for head injury safety standards use only linear head acceleration, and are based on human cadaver skull fracture and animal data.^20^,^24^,^56^ However, more recently it has been shown that the combination of linear and angular head acceleration is a good predictor of concussion, and that helmets reduce both linear and angular acceleration.^28^,^49^,^58^ Given the fact that all head impacts have both linear and rotational acceleration components, future helmet evaluation should quantify injury risk using both linear and rotational head kinematics.

The objective of this study is to describe the development of a new evaluation system for hockey helmets. The evaluation system will provide a quantitative measure of the ability of individual helmets to reduce the risk of concussion. Building on the framework of Football STAR, Hockey STAR will define laboratory test conditions weighted to represent how often hockey players experience similar impacts.

## Methods

### Hockey STAR Equation

The Football STAR equation was developed to identify differences in the ability of football helmets to reduce concussion risk.^48^ The equation represents the predicted concussion incidence for a football player over one season. This predictive value is determined from laboratory tests with a helmeted headform to simulate head impacts at different locations and energy levels. Each laboratory condition is associated with the number of times that type of impact would occur over one season (exposure), and the probability that a concussion would occur due to the resultant head acceleration during each test (risk). In the Football STAR equation (Eq. 1), *L* represents the impact location of front, side, top, or back; *H* represents the drop height of 60, 48, 36, 24, or 12 in; *E* represents the exposure as a function of location and drop height, and *R* represents risk of concussion as a function of linear acceleration (*a*).

1 $$ {\text{Football}}\;{\text{STAR}} = \mathop \sum \limits_{L = 1}^{4} \mathop \sum \limits_{H = 1}^{5} E\left( {L,H} \right)\,*\,R\left( a \right) $$

A similar equation is presented for Hockey STAR, with several important modifications (Eq. 2). The risk function now incorporates both linear and rotational acceleration since all head impacts result in both, and the combination has been shown to be predictive of concussion.^49^,^58^ The exposure component was modified to reflect data collected from hockey players that consisted of both linear and rotational acceleration. In the Hockey STAR equation, *L* represents the head impact locations of front, side, top, or back; *θ* represents different impact energy levels defined by the angle of the pendulum arm used to impact the head; *E* represents exposure, or the number of times per season a player is expected to experience an impact similar to a particular testing condition as a function of location and impact energy; and *R* is the risk of concussion as a function of linear (*a*) and angular (*α*) head acceleration. The exposure and risk components of the equation are described in later sections.

2 $$ {\text{Hockey}}\;{\text{STAR}} = \mathop \sum \limits_{L = 1}^{4} \mathop \sum \limits_{\theta = 1}^{3} E\left( {L,\theta } \right)\,*\,R(a,\alpha ). $$

The laboratory testing matrix includes 3 impact energy levels and 4 impact locations, for a total of 12 testing conditions per helmet. In practice, two helmets of every model will be purchased. Each of these helmets will be tested in the 12 conditions twice for a total of 48 tests per helmet model. The two acceleration values for each helmet’s test conditions will then be averaged for each impact condition prior to using the risk function to determine probability of concussion. Concussion risks will then be multiplied by the exposure values for each impact condition to determine incidence values. All incidence values are then aggregated to calculate a Hockey STAR value for each helmet. The Hockey STAR values for each helmet will then be averaged to determine a helmet model’s overall Hockey STAR value.

### Hockey Head Impact Exposure

Head impact exposure is defined here as the number of impacts a player experiences over one season of play. Data from two different studies were utilized to determine the median number of impacts per season over a broader population of males, females, and youth ice hockey players. Wilcox *et al*. collected data from both male and female National Collegiate Athletic Association (NCAA) ice hockey teams over three seasons from 2009 to 2012 using helmet-mounted accelerometer arrays.^57^ Using the same instrumentation, Mihalik *et al*. collected data from a population of male Bantam (13–14 years old) and Midget (15–16 years old) players over 2 years.^39^ These accelerometer arrays have previously been described in detail, but briefly, each helmet contains six single-axis linear accelerometers that are oriented tangentially to the head and integrated into foam inserts which allow the sensors to maintain contact with the head during impact.^25^ The median number of head impacts per player per season experienced by collegiate athletes was 287 for males and 170 for females.^57^ The median number of impacts per player per season for youth athletes was 223.^39^ The median values for each population were averaged to determine an overall exposure of 227 impacts. This value was used to represent the total number of impacts for one player over one season. The exposure value was further defined by impact location and severity as described below.

Data collected with the helmet-mounted accelerometer arrays was used to map on-ice player impact exposure to lab conditions.^7^,^57^ Data from two male and two female NCAA ice hockey teams as well as one male and one female high school team were included. The data were scaled to reduce measurement error using a relationship determined from correlating resultant head accelerations calculated from the helmet instrumentation to a reference measurement in an instrumented dummy headform during controlled laboratory impact tests.^3^

The helmet data were then stratified by impact location. The locations are defined by the azimuth and elevation of the impact vector and are generalized into bins representing the front, right, left, back and top of the head.^23^ The front, right, left, and back consist of impacts with an elevation less than 65°, and are divided equally into 4 bins that are centered on the intersection of the midsagittal and coronal planes, but offset by 45°. The remaining impacts greater than 65° in elevation are grouped as top impacts. The exposure for each impact location was weighted by how often they occur in data collected in the literature.^7^,^25^,^39^,^57^ The front, side (left and right combined), and back were approximately 30% each, with the remaining 10% of impacts to the top of the head. These values were used to weight exposure by impact location.

### Hockey Helmet Impact Device

The next step in defining exposure was to transform on-ice player head acceleration data distributions to impact conditions in the lab. To do this, a series of impact tests were performed over a range of input energies using a custom impact pendulum to map laboratory-generated head accelerations to those measured on-ice directly from hockey players. The impact pendulum system used for these tests, impact locations evaluated, and methods for the acceleration transformation are described in detail below.

A pendulum was chosen due to increased repeatability and reproducibility when compared with other head impact methods.^45^ The pendulum arm is composed of 10.16 × 5.08 cm rectangular aluminum tubing with a 16.3 kg impacting mass at its end. The length of the pendulum arm from the center of its pivot point to the center of its impacting mass is 190.5 cm. The pendulum arm has a total mass of 36.3 kg and a moment of inertia of 72 kg m^2^. The impacting mass accounts for 78% of the total moment of inertia. The nylon impactor face has a diameter of 12.7 cm, which is flat and rigid in an effort to maximize repeatability and reproducibility of the tests. Furthermore, a rigid impacting face was chosen due to rigid surfaces in hockey, and to avoid impactor compliancy masking differences between helmets in comparative testing.^47^

**Figure 1:**
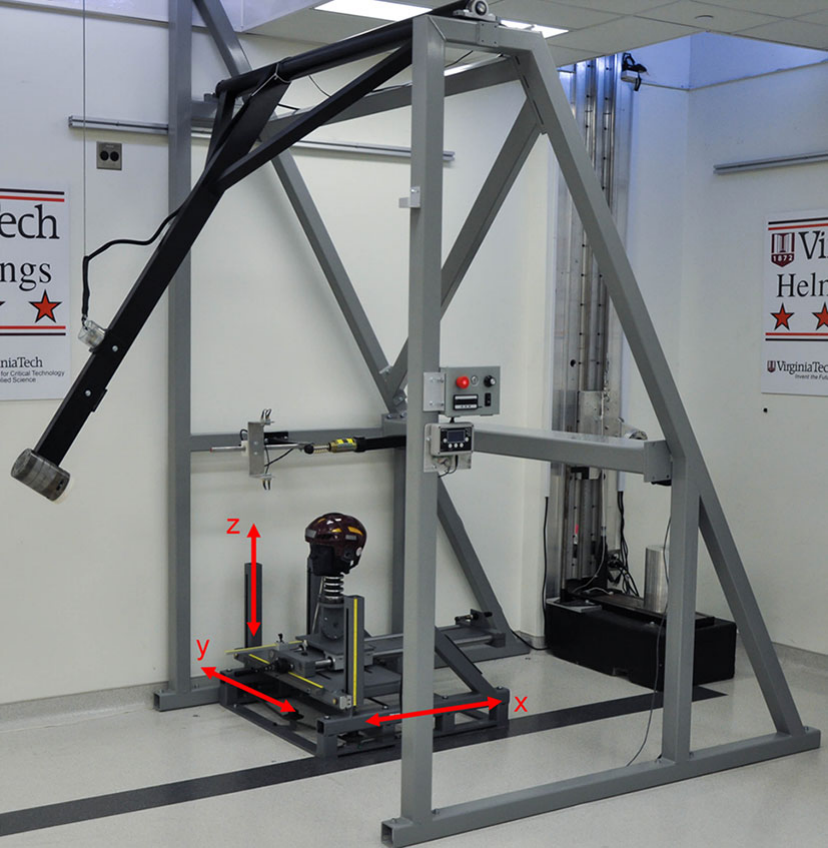
The custom impact pendulum device was used to strike a NOCSAE headform mounted on a Hybrid III 50th percentile neck. The head and neck were mounted on a sliding mass that simulates the effective mass of the torso during impact. The slide table has 5 degrees of freedom so that any location on the helmet could be impacted: translation along the *x* axis, translation along the *y* axis, translation along the *z* axis, rotation about the *y* axis, and rotation about the *z* axis.

The pendulum impactor strikes a medium NOCSAE headform, which is mounted on a Hybrid III 50th percentile neck (Fig. 1). The NOCSAE headform was used to provide the most realistic fit between helmet and headform.^11^ A custom adaptor plate was used to mate the NOCSAE headform to the Hybrid III neck while keeping the relative locations of the occipital condyle pin and headform center of gravity (CG) as close as possible to that of the Hybrid III 50th percentile male head and neck assembly. Material was removed from the underside of the headform to optimize the position of the occipital condyle and accommodate the neck. The adaptor plate’s mass was equal to the material removed. Although these distances matched exactly in the anterior-posterior and medial–lateral directions, the NOCSAE CG was 22 mm superior relative to the Hybrid III CG. The head and neck assembly are mounted on a sliding mass intended to simulate the effective the mass of the torso during impact. This sliding mass is part of a commercially available linear slide table that is commonly used for helmet impact testing (Biokinetics, Ottawa, Ontario, Canada). Contrary to most helmet drop test rigs, this system allows for linear and rotational motion to be generated during impact. To measure the kinematics resulting from impact, the headform was instrumented with a 6 degree of freedom sensor package consisting of 3 accelerometers and 3 angular rate sensors (6DX-Pro, DTS, Seal Beach, CA).

**Figure 2:**
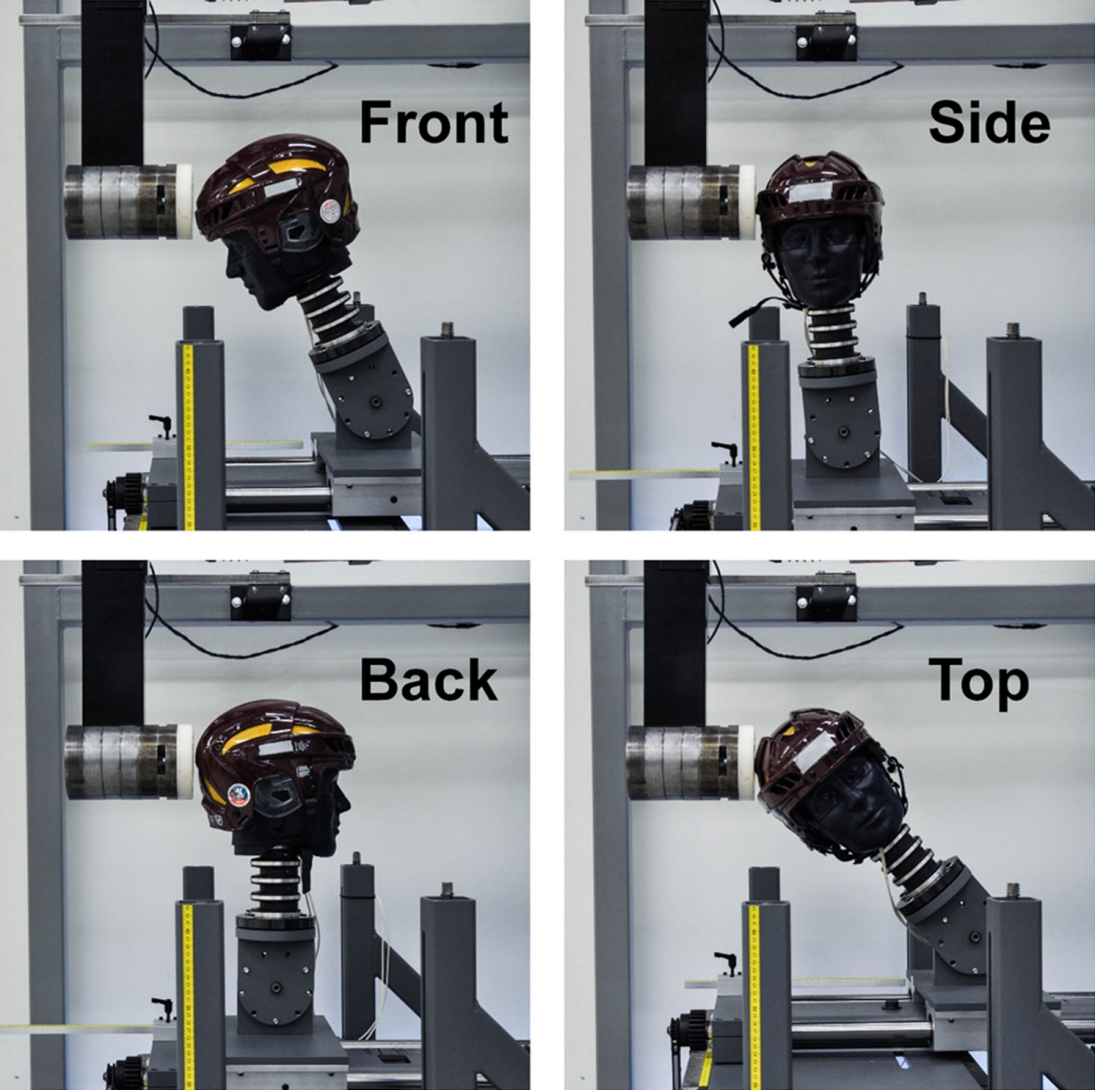
Photographs of the front, side, back, and top impact locations used to assess helmet performance. The side and top impact locations are non-centric, meaning the direction of force is not aligned with the CG of the headform; while the front and back impact locations are centric.

The front, side, back, and top of the headform were chosen to impact in laboratory tests (Fig. 2). In order to account for a wider array of impact types, two of the locations were centric, or aligned with the CG of the headform (front and back), and two were non-centric (side and top). These locations resulted in some impacts with higher rotational components for a given linear acceleration than others, which was quantified by the effective radius of rotation at each condition. Effective radius of rotation was defined as the quotient of peak linear acceleration and peak rotational acceleration. Table 1 specifies the impact locations using measurement markings provided on the commercially available linear slide table.

**Table 1:** Measurement markings and angles of rotation on the linear slide table for each impact location tested.

	*Y* translation (cm)	*Z* translation (cm)	*Y* rotation (°)	*Z* rotation (°)
Front	40.3	8.9	25	0
Side	36.9	3.5	5	80
Top	42.7	13.5	40	90
Back	40.3	4.9	0	180

### Mapping Exposure Data to Laboratory System

A series of tests were performed to map the on-ice helmet data to laboratory pendulum impacts. For these tests, the NOCSAE headform was fitted with a size medium CCM Vector V08 helmet (Reebok-CCM Hockey, Inc., Montreal, Canada). The V08 model was chosen because it was one of the helmet types worn by instrumented players to generate head impact exposure data.^57^ The linear acceleration and angular rate data were collected at a sampling rate of 20,000 Hz. Linear acceleration data were filtered to CFC 1000 Hz according to SAE J211, while angular rate data were filtered to CFC 155. Angular acceleration was calculated by differentiating the angular rate data. All data were then transformed to the CG of the headform. Three V08 helmets were tested, with each impacted from pendulum arm angles of 20°, 30°, 40°, 50°, 60°, 70°, 80°, and 90° at each of the four locations defined above, resulting in 96 impact tests.

After determining the total impact exposure per player per season and stratifying the on-ice helmet data by impact location, the data were transformed to laboratory impact conditions. To do this, the on-ice data for each location were reduced to include only impacts with effective radii of rotation in the range of corresponding laboratory impacts. Within these constraints, the on-ice head acceleration distributions were related to impact conditions in the lab. Bivariate empirical cumulative distribution functions (CDF) comprised of peak linear and peak rotational head accelerations were computed for on-ice data within each impact location’s constraints. The CDFs were defined by determining the percentage of impacts less than or equal to each impact’s peak linear and peak rotational acceleration. Using the location-specific CDFs, the percentile impact for each pendulum impact energy was determined by relating peak linear and peak rotational acceleration average values generated from each laboratory condition. Through this process, location-specific impact energy CDFs were determined for each population (male collegiate, female collegiate, male high school, and female high school). The 4 resulting impact energy CDFs were then averaged for equal weighting between populations.

Low, medium, and high impact energy conditions were set prior to computing the weighting used in the Hockey STAR formula. These conditions were chosen to be representative of a span of impacts severities that encompass both sub-concussive and concussive head impacts, and are defined by pendulum arm angles of 40° (low), 65° (medium), and 90° (high). Weightings to be used for the Hockey STAR test configurations were determined by setting bounds on the impact energy CDFs midway between each test angle. For each location, the percentage of impacts below 52.5° was defined as the low energy condition, the percentage of impacts between 52.5° and 77.5° was defined as medium energy condition, and the percentage of impacts greater than 77.5° was defined as the high energy condition. The weightings for each test configuration were then computed by multiplying these percentages by the total number of head impacts that the average hockey player sustains at each location.

### Injury Risk Function

The risk function used in Hockey STAR was updated to incorporate both linear (a) and rotational head acceleration (α) components (Eq. 3). Development of the combined risk function for concussion has previously been described.^49^

3 $$ R(a,\alpha ) = \frac{1}{{1 + e^{ - ( - 10.2 + 0.0433*a + 0.000873*\alpha - 0.000000920*a\alpha )} }} $$

In short, the risk function was developed using data collected from high school and collegiate football players. A multivariate logistic regression analysis was used to model risk as a function of linear and rotational head acceleration. There is an interaction term because linear and rotational acceleration are correlated. This risk function is unique in that it accounts for the under-reporting of concussion in the underlying data used to develop the curve.^33^,^35^ The predictive capability of the risk function was found to be good using NFL head impact reconstructions in addition to the impacts used to generate the function.

### Exemplar Hockey Helmet Tests

Three exemplar helmets are used to demonstrate Hockey STAR. Each helmet was tested in 12 impact conditions: 4 locations with 3 impact energies per location. Pendulum arm angles of 40°, 65°, and 90° were tested, which equate to impact velocity of 3, 4.6, and 6.1 m/s. These illustrative tests differ from actual Hockey STAR tests in that only one helmet per model was tested, and each test configuration was only tested once. In practice, each test condition would be tested twice for each helmet, and acceleration values in each condition would be averaged before calculating risk. Hockey STAR values for the two helmets of each model are averaged to determine a helmet model’s overall Hockey STAR value. For demonstrative purposes, two hockey helmets and one football helmet were tested under these conditions and Hockey STAR values calculated.

## Results

### Mapping Exposure Data to Laboratory System

**Figure 3:**
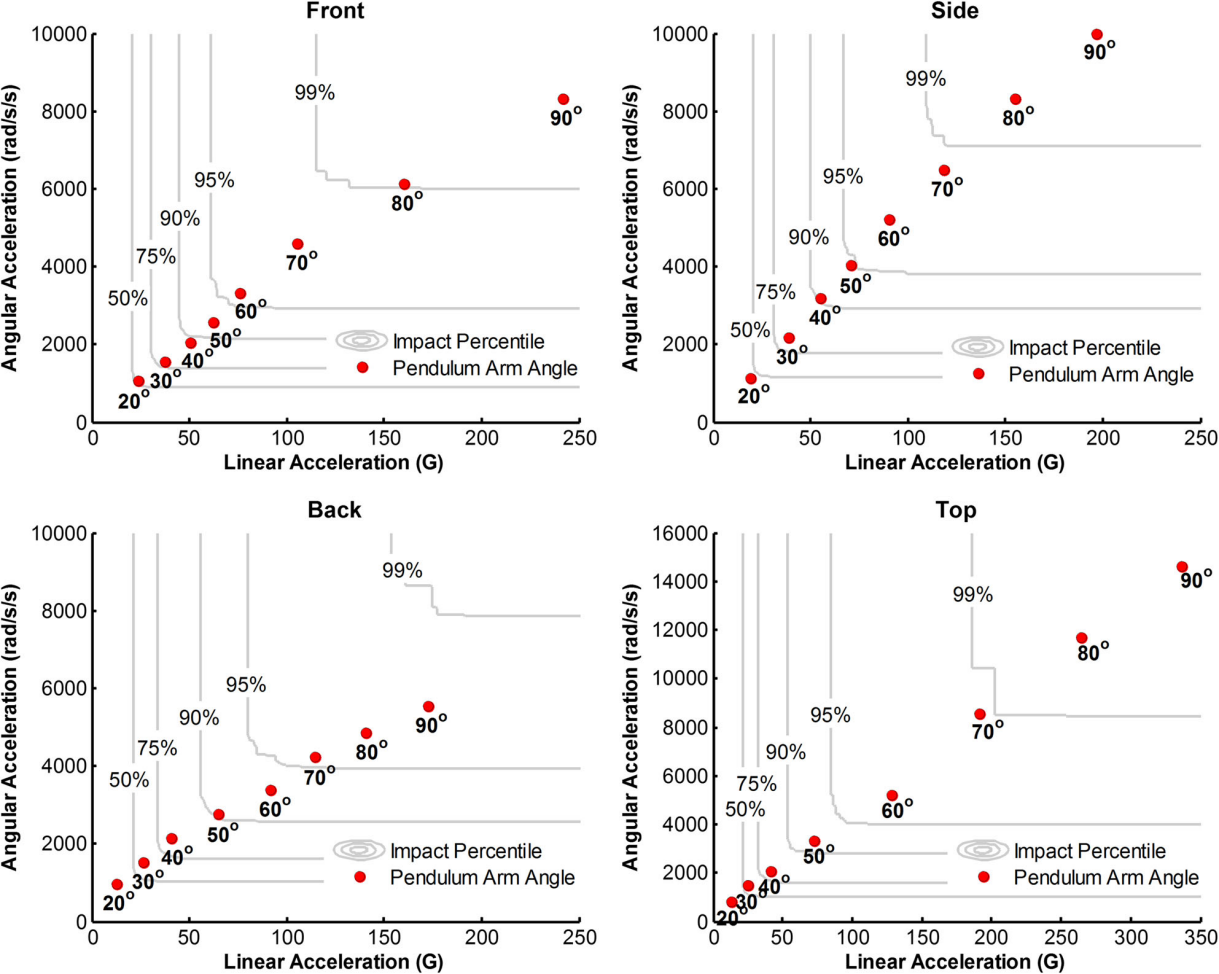
Peak linear and rotational head acceleration values generated during the pendulum tests are overlaid on the bivariate CDFs for each impact location. These plots relate laboratory impact energies to on-ice head impact data and were used to define head impact distributions as a function of impact energy. Where a given impact energy (pendulum arm angle) fell within the distributions varied by impact location. While these plots only illustrate this for male collegiate hockey, this was done for each of the 4 hockey player populations in which on-ice data were previously collected.

Bivariate CDFs for linear and rotational accelerations experienced by male collegiate hockey players are shown in Fig. 3 for each impact location. Peak linear and rotational head acceleration values generated during the pendulum tests are overlaid on the CDFs to illustrate how the laboratory tests relate to the on-ice head impact distributions. Constant impact energies varied in percentile by impact location. For example, releasing the pendulum arm from 40° was representative of the 88.2 percentile impact to the front location, 90.4 percentile impact to the side location, 81.4 percentile impact to the back location, and 80.7 percentile impact to the top location. This demonstrates that higher head accelerations were more commonly associated with back and top impact locations in the on-ice helmet data. The tails of these right-skewed distributions exhibited similar trends. Releasing the pendulum arm from 70° was representative of the 98.2 percentile impact to the front location, 98.6 percentile impact to the side location, 95.5 percentile impact to the back location, and 98.9 percentile impact to the top location.

**Figure 4:**
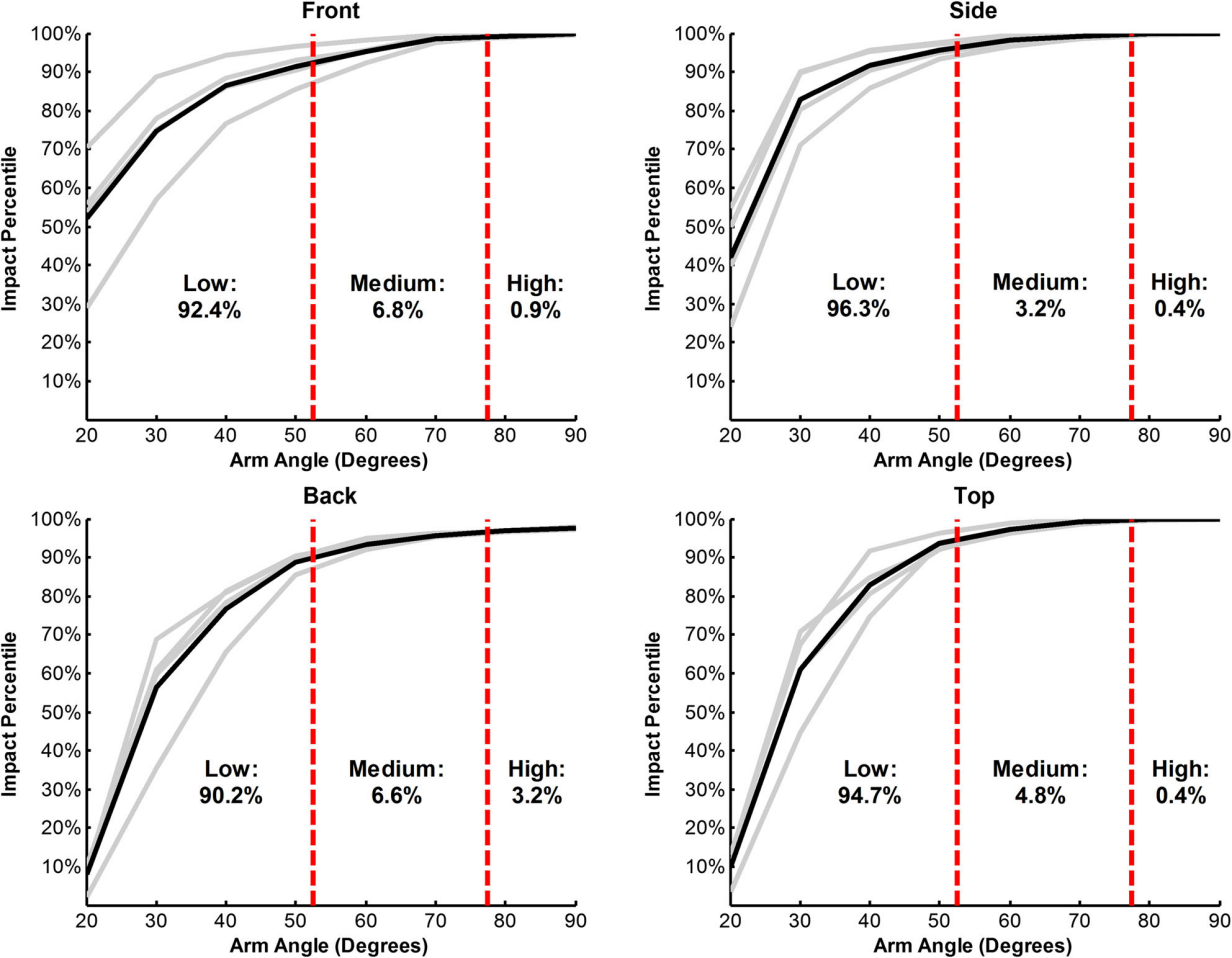
Impact energy CDFs for each impact location resulting from the transformation of on-ice data to laboratory impact conditions. The gray lines represent impact energy CDFs for each population and the black line is the equal-weight average of the four populations. The dashed red lines show the bounds used to determine the percentage of impacts at each location associated with the low, medium, and high energy impact conditions. This analysis was used to define the exposure weightings for each impact configuration in the Hockey STAR formula.

On-ice head acceleration distributions were transformed to impact energy distributions (represented by pendulum arm angle) by determining the percentage of on-ice data that fell below each energy for each impact location. This process was done for each population (male and female collegiate, male and female high school). Resulting impact energy CDFs were then averaged to determine an overall impact energy CDF that gave equal weighting to each population (Fig. 4). The impact energy CDFs were related to generalized impact energy conditions: a low energy condition (40° pendulum arm angle), a medium energy condition (65° pendulum arm angle), and a high energy condition (90° pendulum arm angle). For all locations, the low energy condition accounts for greater than 90% of head impacts. The medium energy condition ranged between 3.2 and 6.8% of impacts for each condition. The high energy condition generally accounted for less than 1% of impacts for each location, with the exception of the back location. From this analysis, weightings were determined for each laboratory impact condition based on how frequently a player might sustain a similar impact (Table 2). Summating these laboratory condition-specific exposure values results in the 227 head impacts that the average player experiences throughout a season of hockey.

**Table 2:** Mapping of on-ice head impact exposure to generalized laboratory test conditions.

	40°	65°	90°	Total
Front	62.9	4.6	0.6	68.1
Side	65.6	2.2	0.3	68.1
Top	21.5	1.1	0.1	22.7
Back	61.4	4.5	2.2	68.1
Total	211.4	12.4	3.2	227

Each impact configuration was related to a number of impacts that the average player experiences during a season of play. These numbers represent the exposure weightings for each test condition in the Hockey STAR formula

### Exemplar Hockey Helmet Tests

Three helmets were evaluated with the Hockey STAR evaluation methods described above: two hockey helmets and one football helmet. The detailed results for each helmet are shown in Tables 3, 4, and 5. Hockey STAR values were 7.098 for hockey helmet A, 12.809 for hockey helmet B, and 1.213 for the football helmet. Lower STAR values equate to lower risk of concussion. Given the assumptions that all players experience an identical head impact exposure to that which was modeled and had the same concussion tolerance to head impact, these STAR values suggest that the concussion rate for players in hockey helmet A would be 44.6% less than that of players in hockey helmet B. Comparing the hockey helmets to the football helmet, players in the football helmet would experience concussions rates 82.9% less than players in hockey helmet A and 90.5% less than players in hockey helmet B.

**Table 3:** Hockey STAR evaluation of hockey helmet A helmet with resultant peak linear (*a*) and angular (*α*) acceleration, corresponding risk of injury, and season exposure for each condition to calculate incidence.

Impact location	Angle (°)	Peak *a* (g)	Peak *α* (rad/s^2^)	Risk of injury (%)	Exposure per season	Incidence per season
Front	40	64	2154	0.34	62.9	0.213
Front	65	108	3591	5.94	4.6	0.273
Front	90	168	6680	86.57	0.6	0.519
Side	40	71	4220	2.39	65.6	1.568
Side	65	124	7149	64.74	2.2	1.424
Side	90	176	9370	98.34	0.3	0.295
Top	40	37	2590	0.16	21.5	0.035
Top	65	103	6061	26.23	1.1	0.289
Top	90	263	12,666	99.99	0.1	0.100
Back	40	41	2020	0.12	61.4	0.072
Back	65	111	4345	11.43	4.5	0.514
Back	90	169	6076	81.60	2.2	1.795

					STAR	7.098

The resulting Hockey STAR value is 7.098

**Table 4:** Hockey STAR evaluation of hockey helmet B with resultant peak linear (*a*) and angular (*α*) acceleration, corresponding risk of injury, and season exposure for each condition to calculate incidence.

Impact location	Angle (°)	Peak *a* (g)	Peak *α* (rad/s^2^)	Risk of injury (%)	Exposure per season	Incidence per season
Front	40	64	2570	0.48	62.9	0.299
Front	65	87	3819	3.21	4.6	0.148
Front	90	164	6333	81.58	0.6	0.489
Side	40	74	5037	5.04	65.6	3.305
Side	65	115	8254	75.17	2.2	1.654
Side	90	155	10,189	98.12	0.3	0.294
Top	40	66	3869	1.47	21.5	0.315
Top	65	124	7001	61.60	1.1	0.678
Top	90	163	9548	97.72	0.1	0.098
Back	40	56	3448	0.71	61.4	0.435
Back	65	135	6647	65.27	4.5	2.937
Back	90	178	9073	98.07	2.2	2.158

					STAR	12.809

The resulting Hockey STAR value is 12.809

**Table 5:** Hockey STAR evaluation of a football helmet with resultant peak linear (*a*) and angular (*α*) acceleration, corresponding risk of injury, and season exposure for each condition to calculate incidence.

Impact Location	Angle (°)	Peak *a* (g)	Peak *α* (rad/s^2^)	Risk of injury (%)	Exposure per season	Incidence per season
Front	40	37	1787	0.08	62.9	0.052
Front	65	76	2679	0.84	4.6	0.039
Front	90	115	3646	8.21	0.6	0.049
Side	40	35	2210	0.11	65.6	0.072
Side	65	64	3940	1.47	2.2	0.032
Side	90	122	7120	61.95	0.3	0.186
Top	40	32	1965	0.08	21.5	0.017
Top	65	67	3554	1.20	1.1	0.013
Top	90	100	4622	9.28	0.1	0.009
Back	40	44	2177	0.16	61.4	0.096
Back	65	78	3886	2.37	4.5	0.107
Back	90	109	5644	24.60	2.2	0.541

					STAR	1.213

The resulting Hockey STAR value is 1.213

## Discussion

The purpose of this paper is to introduce a new evaluation system for hockey helmets that can provide information to consumers on the relative performance of different helmets. Hockey STAR is in no way meant to diminish the importance of, or replace, the current ASTM standards enforced by HECC. Since the introduction of these standards and other rule changes in the game, the rate of catastrophic head injuries has greatly decreased.^6^ The standards also require important specifications regarding the elongation of the chin strap and appropriate area of coverage of helmets. The Hockey STAR evaluation system intends to only test hockey helmets that have already been certified by HECC. HECC and other helmet certifications are analogous to the Federal Motor Vehicle Safety Standards (FMVSS) and regulations which have pass/fail standards. These standards provide baseline safety requirements that are crucial for protecting drivers. The New Car Assessment Program (NCAP) developed by the National Highway Traffic Safety Administration (NHTSA) augments the existing standards by providing consumers with a rating system to help guide their selections.^26^,^29^ Hockey and Football STAR serve the same purpose as NCAP: to provide additional information to consumers after the minimum safety requirements have been met through certification.

### Advances from Football STAR

Like Football STAR, Hockey STAR is based on two fundamental principles: (1) helmets that lower head acceleration reduce concussion risk and (2) each test is weighted based on how often players experience similar impacts. An Institute of Medicine (IOM) report on sport-related concussion in youth reviewed Football STAR and characterized it as a theoretically grounded approach to evaluating helmet protection that is based on sound principles.^38^ However, the report also noted that adding rotational acceleration to the methodology would increase its wide-spread application. Considering this recommendation, Hockey STAR was developed to evaluate helmets using both linear and rotational head acceleration. This addition contributed to the unique head impact exposure analysis in Hockey STAR. The exposure distributions used to weight each impact configuration included both linear and rotational head acceleration from collegiate hockey players.^57^ The total number of impacts over one season was also an average of impacts experienced by youth boy’s and collegiate men’s and women’s hockey, since the same helmet models are used for all ages and genders with variations only in helmet size.^39^,^57^ This is one of two key differences between Football STAR and Hockey STAR.

The second key difference is that Hockey STAR accounts for a higher underreporting rate of concussion than Football STAR. The bivariate risk function was developed with the assumption that only 10% of concussions sustained by players are diagnosed by physicians.^33^,^49^ In contrast, the Football STAR risk function assumes that 50% of concussions sustained by players are diagnosed by physicians.^35^,^48^ Recent studies have suggested that the underreporting rate may be much greater than 50%, and have even suggested that structural changes occur as a result of cumulative head impact exposure in the absence of diagnosed concussion.^4^,^13^,^54^ Because the risk function utilized by Hockey STAR assumes that 90% of concussions go unreported, the Hockey STAR values are not anticipated to be predictive of the number of diagnosed concussions sustained by hockey players, but rather the total number of injuries sustained, diagnosed and undiagnosed.

### Biofidelity of Impact Model

The biofidelity of the impact model used for Hockey STAR was ensured through appropriate headform selection and comparison of acceleration traces with other data collected from hockey players. The NOCSAE headform was chosen because of its superior helmet fit at the base of the skull, and around the jaw, cheeks, and chin compared to that of the Hybrid III headform.^11^ A helmet that does not fit properly can shift on the head during tests, and if the contact area of the helmet padding with the headform varies from what is realistic, the effective stiffness of the padding will vary, potentially resulting in a mischaracterization of a helmet’s energy management capabilities.

**Figure 5:**
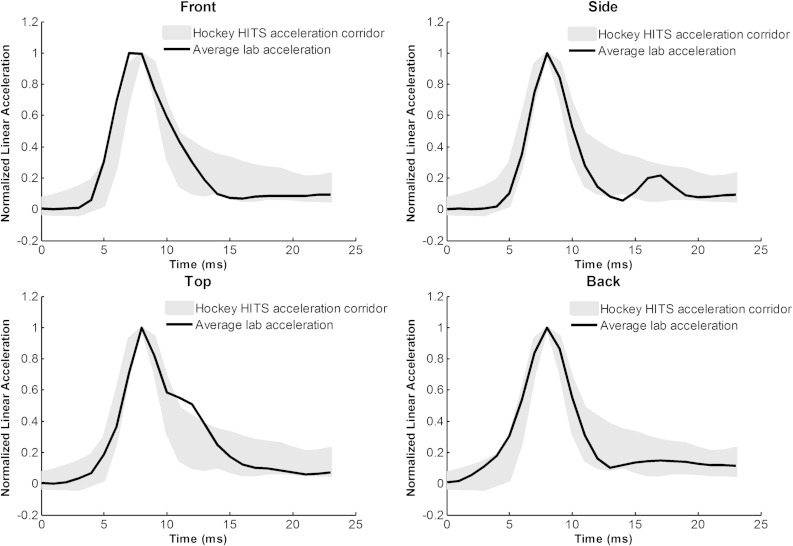
Average acceleration traces from the laboratory pendulum tests were compared to corridors developed from on-ice volunteer data by impact location. The head impact response of the laboratory tests closely matches that which was measured directly from hockey players, suggesting the impact system generates a biofidelic response.

**Figure 6:**
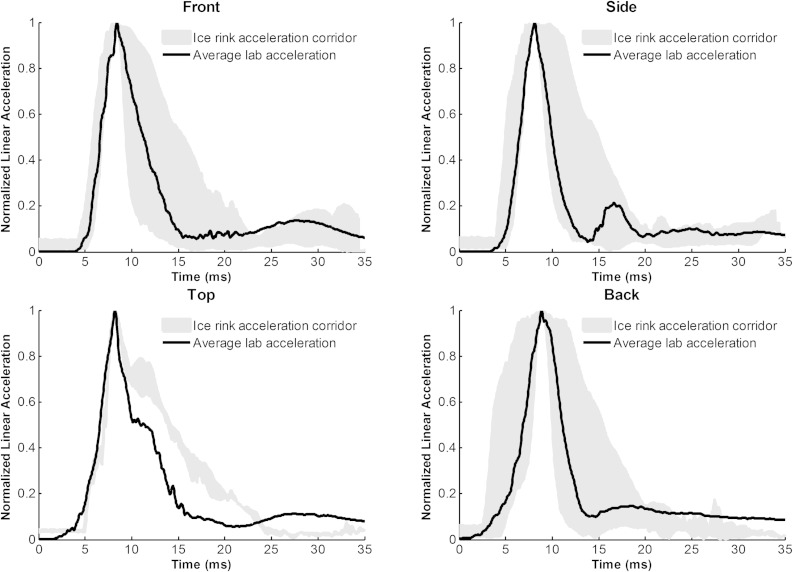
Head impact responses generated in the lab were also compared to dummy head impacts collected at an ice rink. Here, average acceleration traces from the laboratory pendulum tests were compared to corridors developed from controlled dummy head impacts to the boards, glass, and ice at an ice rink. The head impact response of the laboratory tests closely matches that which was measured at the ice rink, which further suggests that impact system generates a biofidelic response.

The headform responses generated from pendulum impacts in the lab were compared to on-ice data by generating corridors from both on-ice player data and ice rink testing with a Hybrid III head (Figs. 5, 6). The lab impacts fell within the response corridors generated from both datasets with the exception of the top impacts in the lab compared with the top impacts from ice rink testing. There are two reasons for this difference. The first is that the top impacts for the ice rink testing were pure axial loading to the top of the headform, while the Hockey STAR top location is non-centric and meant to generate rotational acceleration. The second reason is that the ice condition was not tested for the top location on the ice rink, so only boards and glass responses are averaged. These impacts are longer in duration and not representative of the full spectrum of impacts seen by ice hockey players. Overall, this analysis provides further evidence that the laboratory testing is representative of head impacts in hockey.

### Implementing Hockey STAR

Given that there are 32 helmets currently on the market, a total of 1536 tests are required to evaluate all hockey helmets using the proposed protocol. While this methodology proposes a reasonable number of tests to evaluate helmets, there are practical limitations to the number of tests that can be run. For this reason, there are other variables that have been considered and researched. For example, helmet temperature is not varied in this protocol. We performed a study investigating the temperature inside football helmets during games.^50^ When a player wears a helmet, the temperature of the padding will approach that of the head. For this reason, and that fact that testing multiple temperatures could double or triple the number of tests, helmet temperature is not varied in the Hockey STAR protocol. Additionally, Hockey STAR does not evaluate helmets with a facemask on. There are a number of facemask configurations that can be used on a helmet. These include full cage facemasks and clear visors. Testing in the lab demonstrated that the facemask does not significantly affect either linear or rotational head acceleration, with differences less than 2%. This suggests that hockey helmet performance is not influenced by the presence of a facemask, and that testing with and without facemasks is not necessary. In short, there are a near-infinite number ways to test a helmet, but there are practical limitations to the number of tests used to evaluate products.

### Star Rating Thresholds

The hockey star methodology will ultimately be used to apply star ratings to hockey helmets, which allows consumers to easily compare overall helmet performance between models. While this is already being done with football helmets, the STAR value thresholds used to determine the star ratings of football helmets cannot simply be applied to hockey helmet evaluations due to a number of key differences in the Hockey STAR and Football STAR formulas. The impact exposure weightings are specific to each sport, the test conditions differ, and a more conservative risk function is used in the Hockey STAR methodology. Current football helmet ratings were re-analyzed using a similarly conservative risk function for linear head acceleration.^51^ The differences in test conditions were also accounted for by comparing the results of the exemplar football helmet tested under Hockey STAR conditions to the results of the same helmet tested with Football STAR. Proposed star rating thresholds for Hockey STAR are based on these equivalent values (Table 6).

**Table 6:** Comparison of the proposed Hockey STAR rating thresholds to the current thresholds used in Football STAR and Hockey STAR thresholds that are equivalent to current Football STAR thresholds using the proposed methodology.

Star rating	Current football STAR	Equivalent Hockey STAR	Proposed Hockey STAR
5	0.300	1.463	1.500
4	0.400	2.069	2.000
3	0.500	2.676	2.500
2	0.700	3.889	4.000
1	1.000	5.708	6.000

To earn a number of stars, a helmet’s STAR value must be below the specified threshold.

### Exemplar Hockey STAR Results

For the three helmets tested using the Hockey STAR methodology, the Hockey STAR values were 7.098, 12.809, and 1.213 for helmet A, helmet B, and the football helmet, respectively. These values are related to the relative risk of concussion, such that a player wearing helmet A would be 44.6% less likely to sustain a concussion than a player wearing helmet B if both players had the same head impact exposure over one season. Similarly, if a player wore the football helmet and also had the same head impact exposure, that player would be 82.9% less likely to sustain a concussion than a player wearing helmet A, and 90.5% less likely than a player wearing helmet B. Again, it is important to note that these STAR values are not representative of the number of diagnosed concussions players will experience, but rather an overall estimate of undiagnosed and diagnosed injuries combined. While these values are tied to concussion risk, ultimately the rating system identifies helmets that best reduce head acceleration throughout the range of head impacts that hockey players experience.

Given the proposed thresholds outlined in Table 6, helmets A and B would not be recommended and the football helmet would receive a 5 star rating. The disparity in performance between the football and hockey helmets can be attributed to the differences in padding and design for energy attenuation. Specifically, the football helmet has a greater offset, which allows more compression during impact when modulating the impact energy. This enables the padding system to compress on lower severity impacts and not bottom out on higher severity head impacts. The hockey helmets’ padding systems are much thinner, restricting the ability to reduce head acceleration throughout the full range of head impacts experienced by players.

## Conclusions

This paper presents a novel methodology for comparing the performance of different hockey helmets. The methods are comparable to the existing Football STAR rating system, however the equation has been updated to include both linear and rotational acceleration. The exposure and testing conditions were also modified to represent the number and type of head impacts experienced by hockey players. A new impact pendulum was designed and built for laboratory testing, and the biofidelity of the system was ensured by comparison with on-ice player data and other testing methods. Given that Hockey STAR will be used to rate hockey helmets, exemplar tests of existing helmets were performed to evaluate and compare of the ability of a small sample of helmets to reduce risk of concussion.

Similar outcomes to those resulting from Football STAR are anticipated for Hockey STAR. Consumers will use the hockey helmet evaluations as a purchasing tool, which will drive manufacturers to advance hockey helmet design to reduce concussion risk. This reduction in concussion risk measured in the lab will translate to hockey players because the laboratory evaluations are representative of head impacts experienced by hockey players.

Finally, it is important to note that no helmet can completely protect a player from all head injuries, and there are always risks associated with playing the sport. The analysis presented here is based on trends and probabilities, but an individual’s risk of concussion may vary with a number of factors such as prior history of head injury or genetic predispositions.

## References

[1] Agel J, Dick R, Nelson B, Marshall SW, Dompier TP (2007). Descriptive epidemiology of collegiate women’s ice hockey injuries: national collegiate athletic association injury surveillance system, 2000–2001 through 2003–2004. J. Athl. Train..

[2] Agel J, Dompier TP, Dick R, Marshall SW (2007). Descriptive epidemiology of collegiate men’s ice hockey injuries: national collegiate athletic association injury surveillance system, 1988–1989 through 2003–2004. J. Athl. Train..

[3] Allison MA, Kang YS, Bolte JHT, Maltese MR, Arbogast KB (2014). Validation of a helmet-based system to measure head impact biomechanics in ice hockey. Med. Sci. Sports Exerc..

[4] Baugh CM, Kiernan PT, Kroshus E, Daneshvar DH, Montenigro PH, McKee AC, Stern R (2015). Frequency of head impact related outcomes by position in NCAA Division I Collegiate Football Players. J. Neurotrauma.

[5] Benson BW, McIntosh AS, Maddocks D, Herring SA, Raftery M, Dvorak J (2013). What are the most effective risk-reduction strategies in sport concussion?. Br. J. Sports Med..

[6] Biasca N, Wirth S, Tegner Y (2002). The avoidability of head and neck injuries in ice hockey: an historical review. Br. J. Sports Med..

[7] Brainard LL, Beckwith JG, Chu JJ, Crisco JJ, McAllister TW, Duhaime AC, Maerlender AC, Greenwald RM (2012). Gender differences in head impacts sustained by collegiate ice hockey players. Med. Sci. Sports Exerc..

[8] Centers for Disease Control and Prevention (2011). Nonfatal traumatic brain injuries related to sports and recreation activities among persons aged ≤19 years—United States, 2001–2009. MMWR Morb. Mortal. Wkly. Rep..

[9] Chamard, E., H. Theoret, E. N. Skopelja, L. A. Forwell, A. M. Johnson, and P. S. Echlin. A prospective study of physician-observed concussion during a varsity university hockey season: metabolic changes in ice hockey players. Part 4 of 4. *Neurosurg. Focus.* 33(6):E4: 1–7, 2012. 10.3171/2012.10.FOCUS12305 23199427

[10] Clay MB, Glover KL, Lowe DT (2013). Epidemiology of concussion in sport: a literature review. J. Chiropr. Med..

[11] Cobb, B. R., A. MacAlister, T. J. Young, A. R. Kemper, S. Rowson, and S. M. Duma. Quantitative comparison of hybrid III and national operating committee on standards for athletic equipment headform shape characteristics and implications on football helmet fit. *Proc. Inst. Mech. Eng. P*. 1754337114548245, 2014.

[12] Covassin T, Elbin RJ, Sarmiento K (2012). Educating coaches about concussion in sports: evaluation of the CDC’s “Heads Up: Concussion in Youth Sports” initiative. J. Sch. Health..

[13] Davenport EM, Whitlow CT, Urban JE, Espeland MA, Jung Y, Rosenbaum DA, Gioia G, Powers AK, Stitzel JD, Maldjian JA (2014). Abnormal white matter integrity related to head impact exposure in a season of high school varsity football. J. Neurotrauma..

[14] Echlin PS (2010). Concussion education, identification, and treatment within a prospective study of physician-observed junior ice hockey concussions: social context of this scientific intervention. Neurosurg. Focus..

[15] Echlin PS, Johnson AM, Riverin S, Tator CH, Cantu RC, Cusimano MD, Taunton JE, Upshur RE, Hall CR, Forwell LA, Skopelja EN (2010). A prospective study of concussion education in 2 junior ice hockey teams: implications for sports concussion education. Neurosurg. Focus..

[16] Echlin, P. S., E. N. Skopelja, R. Worsley, S. B. Dadachanji, D. R. Lloyd-Smith, J. A. Taunton, L. A. Forwell, and A. M. Johnson. A prospective study of physician-observed concussion during a varsity university ice hockey season: incidence and neuropsychological changes. Part 2 of 4. *Neurosurg. Focus.* 33(6):E2: 1–11, 2012. 10.3171/2012.10.FOCUS12286 23199425

[17] Echlin PS, Tator CH, Cusimano MD, Cantu RC, Taunton JE, Upshur RE, Czarnota M, Hall CR, Johnson AM, Forwell LA, Driediger M, Skopelja EN (2010). Return to play after an initial or recurrent concussion in a prospective study of physician-observed junior ice hockey concussions: implications for return to play after a concussion. Neurosurg. Focus..

[18] Echlin PS, Tator CH, Cusimano MD, Cantu RC, Taunton JE, Upshur RE, Hall CR, Johnson AM, Forwell LA, Skopelja EN (2010). A prospective study of physician-observed concussions during junior ice hockey: implications for incidence rates. Neurosurg. Focus..

[19] Funk JR, Rowson S, Daniel RW, Duma SM (2012). Validation of concussion risk curves for collegiate football players derived from hits data. Ann. Biomed. Eng..

[20] Gadd, C. W. Use of a weighted-impulse criterion for estimating injury hazard. In: Proceedings of the 10th Stapp Car Crash Conference. SAE 660793, 1966.

[21] Gavett, B. E., R. A. Stern, and A. C. McKee. Chronic traumatic encephalopathy: a potential late effect of sport-related concussive and subconcussive head trauma. *Clin. Sports Med.* 30(1):179–188, xi, 2011. 10.1016/j.csm.2010.09.007 PMC2995699 21074091

[22] Gennarelli, T., A. Ommaya, and L. Thibault. Comparison of translational and rotational head motions in experimental cerebral concussion. In: Proceedings of the 15th Stapp Car Crash Conference, pp. 797–803, 1971.

[23] Greenwald, R. M., J. T. Gwin, J. J. Chu, and J. J. Crisco. Head impact severity measures for evaluating mild traumatic brain injury risk exposure. *Neurosurgery*. 62(4):789–798; discussion 98, 2008. 10.1227/01.neu.0000318162.67472.ad PMC2790598 18496184

[24] Gurdijan ES, Roberts VL, Thomas LM (1966). Tolerance curves of acceleration and intracranial pressure and protective index in experimental head injury. J. Trauma..

[25] Gwin JT, Chu JJ, McAllister TA, Greenwald RM (2009). In situ measures of head impact acceleration in NCAA Division I men’s ice hockey: implications for ASTM F1045 and other ice hockey helmet standards. J. ASTM Int..

[26] Hackney, J. R. and C. J. Kahane. The new car assessment program: five star rating system and vehicle safety performance characteristics. SAE Technical Paper Series. SAE 851245, 1995.

[27] Hardy WN, Khalil TB, King AI (1994). Literature review of head injury biomechanics. Int. J. Impact Eng..

[28] Hardy WN, Mason MJ, Foster CD, Shah CS, Kopacz JM, Yang KH, King AI, Bishop J, Bey M, Anderst W, Tashman S (2007). A study of the response of the human cadaver head to impact. Stapp Car Crash J..

[29] Hershman, L. I. The US new car assessment program (NCAP): past, present, and future. In: Enhanced Safety of Vehicles, Paper Number 390, pp. 1–13, 2001.

[30] Hootman JM, Dick R, Agel J (2007). Epidemiology of collegiate injuries for 15 sports: summary and recommendations for injury prevention initiatives. J. Athl. Train..

[31] King, A. I., K. H. Yang, L. Zhang, W. Hardy, and D. C. Viano. Is head injury caused by linear or angular acceleration? In: Proceedings of the International Research Conference on the Biomechanics of Impact (IRCOBI), 2003.

[32] Koerte, I. K., D. Kaufmann, E. Hartl, S. Bouix, O. Pasternak, M. Kubicki, A. Rauscher, D. K. Li, S. B. Dadachanji, J. A. Taunton, L. A. Forwell, A. M. Johnson, P. S. Echlin, and M. E. Shenton. A prospective study of physician-observed concussion during a varsity university hockey season: white matter integrity in ice hockey players. Part 3 of 4. *Neurosurg. Focus*. 33(6):E3:1–7, 2012. 10.3171/2012.10.FOCUS12303 PMC5687247 23199426

[33] Langburt W, Cohen B, Akhthar N, O’Neill K, Lee JC (2001). Incidence of concussion in high school football players of Ohio and Pennsylvania. J. Child Neurol..

[34] Macpherson A, Rothman L, Howard A (2006). Body-checking rules and childhood injuries in ice hockey. Pediatrics..

[35] McCrea M, Hammeke T, Olsen G, Leo P, Guskiewicz K (2004). Unreported concussion in high school football players: implications for prevention. Clin. J. Sport Med..

[36] McCrory P, Meeuwisse WH, Aubry M, Cantu B, Dvorak J, Echemendia RJ, Engebretsen L, Johnston K, Kutcher JS, Raftery M, Sills A, Benson BW, Davis GA, Ellenbogen RG, Guskiewicz K, Herring SA, Iverson GL, Jordan BD, Kissick J, McCrea M, McIntosh AS, Maddocks D, Makdissi M, Purcell L, Putukian M, Schneider K, Tator CH, Turner M (2013). Consensus statement on concussion in sport: the 4th international conference on concussion in sport held in zurich, november 2012. Br. J. Sports Med..

[37] McKee AC, Stern RA, Nowinski CJ, Stein TD, Alvarez VE, Daneshvar DH, Lee HS, Wojtowicz SM, Hall G, Baugh CM, Riley DO, Kubilus CA, Cormier KA, Jacobs MA, Martin BR, Abraham CR, Ikezu T, Reichard RR, Wolozin BL, Budson AE, Goldstein LE, Kowall NW, Cantu RC (2013). The spectrum of disease in chronic traumatic encephalopathy. Brain..

[38] Medicine IO, Council NR (2014). Sports-related concussions in youth: improving the science, changing the culture.

[39] Mihalik JP, Guskiewicz KM, Marshall SW, Blackburn JT, Cantu RC, Greenwald RM (2012). Head impact biomechanics in youth hockey: comparisons across playing position, event types, and impact locations. Ann. Biomed. Eng..

[40] Odelgard, B. The development of head, face, and neck protectors for ice hockey players. Safety in ice hockey, ASTM STP 1050. Philadelphia: American Society for Testing and Materials, 1989.

[41] Omalu, B. I., S. T. DeKosky, R. L. Hamilton, R. L. Minster, M. I. Kamboh, A. M. Shakir, and C. H. Wecht. Chronic traumatic encephalopathy in a national football league player: Part II. *Neurosurgery*. 59(5):1086–1092; discussion 92–93, 2006. 10.1227/01.NEU.0000245601.69451.27 17143242

[42] Omalu, B. I., S. T. DeKosky, R. L. Minster, M. I. Kamboh, R. L. Hamilton, and C. H. Wecht. Chronic traumatic encephalopathy in a national football league player. *Neurosurgery*. 57(1):128–134; discussion-34, 2005. 10.1227/01.neu.0000163407.92769.ed 15987548

[43] Ommaya A, Hirsch A (1971). Tolerances for cerebral concussion from head impact and whiplash in primates. J. Biomech..

[44] Pellman, E. J., D. C. Viano, A. M. Tucker, I. R. Casson, and J. F. Waeckerle. Concussion in professional football: reconstruction of game impacts and injuries. *Neurosurgery*. 53(4):799–812; discussion-4, 2003. 10.1093/neurosurgery/53.3.799 14519212

[45] Pellman, E. J., D. C. Viano, C. Withnall, N. Shewchenko, C. A. Bir, and P. D. Halstead. Concussion in professional football: Helmet testing to assess impact performance—part 11. *Neurosurgery*. 58(1):78–96; discussion 78–96, 2006. 10.1227/01.neu.0000196265.35238.7c 16385332

[46] Roberts WO, Brust JD, Leonard B, Hebert BJ (1996). Fair-play rules and injury reduction in ice hockey. Arch. Pediatr. Adolesc. Med..

[47] Rowson S, Daniel RW, Duma SM (2013). Biomechanical performance of leather and modern football helmets: technical note. J. Neurosurg..

[48] Rowson S, Duma SM (2011). Development of the star evaluation system for football helmets: integrating player head impact exposure and risk of concussion. Ann. Biomed. Eng..

[49] Rowson S, Duma SM (2013). Brain injury prediction: assessing the combined probability of concussion using linear and rotational head acceleration. Ann. Biomed. Eng..

[50] Rowson S, Duma SM (2013). The temperature inside football helmets during head impact: a five year study of collegiate football games. Proc. Inst. Mech. Eng. P..

[51] Rowson, S. and S. M. Duma. Virginia tech helmet ratings—adult football helmet ratings—May 2014. http://www.sbes.vt.edu/helmet, 2014

[52] Rowson S, Duma SM, Beckwith JG, Chu JJ, Greenwald RM, Crisco JJ, Brolinson PG, Duhaime AC, McAllister TW, Maerlender AC (2012). Rotational head kinematics in football impacts: an injury risk function for concussion. Ann. Biomed. Eng..

[53] Rowson S, Duma SM, Greenwald RM, Beckwith JG, Chu JJ, Guskiewicz KM, Mihalik JP, Crisco JJ, Wilcox BJ, McAllister TW, Maerlender AC, Broglio SP, Schnebel B, Anderson S, Brolinson PG (2014). Can helmet design reduce the risk of concussion in football?. J. Neurosurg..

[54] Talavage TM, Nauman EA, Breedlove EL, Yoruk U, Dye AE, Morigaki KE, Feuer H, Leverenz LJ (2014). Functionally-detected cognitive impairment in high school football players without clinically-diagnosed concussion. J. Neurotrauma..

[55] Unterharnscheidt, F. J. Translational versus rotational acceleration: animal experiments with measured inputs. In: Proceedings of the 15th Stapp Car Crash Conference. SAE 710880, 1971. 4220356

[56] Versace, J. A review of the severity index. SAE Technical Paper Series. SAE 710881, 1971.

[57] Wilcox BJ, Beckwith JG, Greenwald RM, Chu JJ, McAllister TW, Flashman LA, Maerlender AC, Duhaime AC, Crisco JJ (2014). Head impact exposure in male and female collegiate ice hockey players. J. Biomech..

[58] Zhang L, Yang KH, King AI (2004). A proposed injury threshold for mild traumatic brain injury. J. Biomech. Eng..

